# Relationship Between Physical Characteristics of Cereal Polysaccharides and Soft Tribology—The Importance of Grain Source and Malting Modification

**DOI:** 10.1002/fsn3.4699

**Published:** 2025-01-07

**Authors:** Rolando Cesar Moreno Ravelo, Martina Gastl, Thomas Becker

**Affiliations:** ^1^ Technical University of Munich, TUM School of Life Sciences, Chair of Brewing and Beverage Technology, Group Raw Material Based Brewing and Beverage Technology Freising Germany; ^2^ Research Center Weihenstephan for Brewing and Food Quality Technical University Munich Freising Germany

**Keywords:** β‐glucans, AF4‐MALS‐DRI, arabinoxylans, beer, conformation, dextrins, friction coefficient, molar mass

## Abstract

Starch and non‐starch polysaccharides ((N)SPs) are relevant in cereal‐based beverages. Although their molar mass and conformation are important to the sensory characteristics of beer and non‐alcoholic beer, their triggering mechanism in the mouth is not fully understood. Soft tribology has emerged as a tool to mimic oral processing (drinking). The contribution of each (N)SPs to the friction coefficient can be determined when they are enzymatically isolated and characterized by chromatography techniques. Thus, this work aimed to study the relationship between the physical characteristics of isolated (N)SPs and their possible contribution to oral processing through soft tribology (friction). To accomplish this, this research analyzes the effect of grain source (barley, wheat, and oats) and its modification (by steeping degree at two levels) to the (N)SPs´ physical characteristics in wort produced on a laboratory scale. Different characteristics were present in the (N)SPs due to the grain source and the degree of modification. When comparing the impact of the grain source, the malted oats showed the highest molar masses. A higher modification degree produced smaller and more compact structures except for wheat's arabinoxylans and dextrins. The conformation ratio ($r_{\mathrm{rms}}/r_{\mathrm{hyd}}$) values indicate the existence of sphere and micro‐gel structures within each (N)SPs, with branches in arabinoxylans and dextrins. Subsequently, soft tribology was measured on all the worts and their correlation to the (N)SPs' data was performed by multivariate analysis. The wort produced with high modification grains generated higher friction responses. However, this was only statistically significant in barley samples. The multivariate analysis showed that within the mouth (tongue) velocity, the apparent density of the (N)SPs, and the molar mass of arabinoxylans and β‐glucans may influence the friction response and, hence, the oral processing in the mouth during oral processing (drinking).

## Graphical abstract

The physical characteristics of the main starch and non‐starch polysaccharides in worts were characterized. The lubrication behaviour of the worts was analyzed by soft tribology. The data analysis showed the relationship between the molar mass and conformation of these polysaccharied and lubrication behaviour in laboratory‐produced worts.

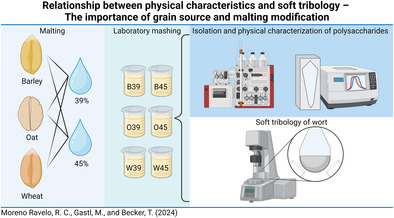

## 1 Introduction

The physical characteristics (molar mass and conformation) of starch and non‐starch polysaccharides ((N)SPs) are relevant for the brewing industry. Although molar mass can also be relevant for filtration (Jin, Speers, and Paulson 2004; Kupetz et al. 2015; Martinez Amezaga, Lataza Rovaletti, and Benítez 2018), previous research has shown the potential of molar mass for sensory characteristics, specifically the *palate fullness* and *mouthfeel* of beer (Langenaeken, De Schutter, and Courtin 2020b; Moreno Ravelo, Gastl, and Becker 2024b; Rübsam, Gastl, and Becker 2013) and non‐alcoholic beer (NAB) (Krebs et al. 2019; Moreno Ravelo et al. 2023). However, the mechanism behind their triggering effect during oral (drinking) processing is unclear. As different polysaccharides show a distinctive lubrication according to their molar mass and conformation, which may greatly vary in beer/NAB, identifying the (N)SPs–lubrication relationship is essential to further understand the human sensory perception from the in‐mouth perspective.

Tribology investigates lubrication, friction, and wear between interacting surfaces in relative motion (Shewan, Pradal, and Stokes 2020). The measurements develop at a constant load at different sliding velocities, which reduces the gap distance (film thickness) between the surfaces to maintain the pressure field (Stokes 2012). Hence, different lubrication regimes can be present during tribological measurements depending on the film thickness, as reviewed elsewhere (Stokes 2012). These regimes are visually displayed as Stribeck curves with parameters such as fluid properties, surface roughness, elasticity, wettability, and adsorption (film formation) influencing the regimes' transition points (Pradal and Stokes 2016).

Soft tribology is a branch of tribology that determines the lubrication while mimicking the interacting surfaces from a biological system of interest. Regarding sensory perception, several surfaces interact in the oral cavity, such as teeth‐teeth, teeth‐palate, teeth‐tongue, and tongue‐palate, to mention some (Stokes, Boehm, and Baier 2013). Among the surfaces available, polydimethylsiloxane (PDMS) has proven to be an analogous surface to the human tongue due to their similarities in hydrophobicity (Carpenter et al. 2019), roughness (Wang, Zhu, and Chen 2021), and elasticity (Biegler et al. 2016). Thus, the food's friction coefficient (FC) can be analyzed from the tongue‐palate cavity perspective when the PDMS is used as a compliant surface.

Polysaccharides, which are broadly present in different food products, are lubricating active substances. Their mechanism of action works by forming a thin film that changes the surface's wettability properties (Shewan, Pradal, and Stokes 2020). Their physical characteristics (e.g., molar mass and conformation) influence the FC of different polysaccharides and food systems. In solutions with similar viscosity, a low molar mass in maltodextrins (800 Da) and carboxymethyl cellulose (90,000 Da) improves the lubrication (decreases the FC values) compared to their high molar masses (13,000 Da and 700,000 Da, respectively) (Ji et al. 2022). Regarding the conformation, a coarser fiber led to higher friction, which also affected the sensory characteristics of fermented yogurt (Kieserling et al. 2019), while the polysaccharide structure influenced the friction response in dairy beverages (Ji et al. 2023). Thus, the physical characteristics of polysaccharides are relevant parameters to assess the soft tribology of food.

In the case of cereal‐based beverages, the grain source is well known to influence the concentration and molar mass of the (N)SPs. The total arabinoxylans (AXs) in the whole grain for barley (B), wheat (W), and oats (O) are 5.98%–8.05%, 4.79%–6.92%, and 2.73%, respectively (Izydorczyk 2009); while the β‐glucans (BGs) content in these grains is in the range of 18.6–53.7 g/kg in B, 17.3–57 g/kg in O, and 1.9–6.7 g/kg in W (Havrlentova and Kraic 2006). The starch amount also differs as W contains 60%–70% of starch (Kim and Kim 2021), 60% on O (Zhang et al. 2021), and 50%–65% on B (Kunze 2004). Although there is not a clear molar mass distinction among the multiple grain sources, precaution is needed when comparing molar mass data assessed by different techniques because the sample handling (e.g., extraction (Tosh et al. 2004) or solubilization (Perez‐Rea, Bergenstahl, and Nilsson 2015)) and the analytical method (e.g., chromatography (White Jr., Hudson, and Adamson 2003), solvents used (Ulmius, Önning, and Nilsson 2012)) affect the obtained molar mass responses. For example, Zhou et al. showed the relevance of the extraction procedure in AXs by comparing enzymatic and alkaline extractions obtaining higher molar masses with the latter (Zhou et al. 2010). On the other hand, fractionating starch with SEC led to lower molar mass values compared to AF4 (Rolland‐Sabate et al. 2011).

Besides the grain source, its modification (kernel transformation) during malting affects the resulting molar masses in the product. The non‐starch polysaccharides (NSPs: AXs and BGs) are degraded upon malting due to the cytolytic activities taking part in the endosperm cell wall (Gastl, Kupetz, and Becker 2021a). Respecting the latter, the steeping degree (SD) and germination time influence the NSPs during malting. Different concentration distributions and molar masses of NSPs are present when employing lower SD (Gastl, Kupetz, and Becker 2021b; Krebs, Becker, and Gastl 2020). Although dextrins (DXs) are mainly affected during the mashing process, changes in their and AXs´ average degree of polymerization due to the germination time have been reported (Langenaeken et al. 2020a). Thus, the combination of different grain sources and their modification through malting regulates the physical responses of (N)SPs in cereal‐based beverages.

Although (N)SPs are relevant for the brewing industry, their molar mass and conformation significance have yet to be thoroughly investigated. Similarly, tribology studies have already been performed on cereal‐based beverages (Fox et al. 2022; Holt and Mills 2023); however, the effect of raw material modification and the role of molar mass and conformation on the lubrication (FC) response have yet to be analyzed. The relationship between raw material modification and FC is important as the physical characteristics of the (N)SPs are dependent on the raw material selection (Marconi et al. 2020) and its modification during malting (Krebs, Becker, and Gastl 2020; Marconi et al. 2014). Therefore, understanding the relationship between molar mass and tribology could allow the modulation and analysis of the beer/NABs from the in‐mouth (drinking) perspective. We hypothesize that different molar masses and conformations of (N)SPs can be present in beer/NABs through raw material sources and malting modification. Consequently, these characteristics influence the sample's lubrication. Therefore, the physical characteristics of the (N)SPs can be used to explain the lubrication response of the samples from the in‐mouth perspective with soft tribology. Laboratory‐produced wort can be a good indicator for quickly assessing the (N)SPs profile as its macromolecular characteristics minorly change after the boiling step (Krebs, Becker, and Gastl 2020). In order to generate a wide variation of molar mass responses, 3 different grain sources (B, W, and O) modified at 2 levels (parameter SD) were employed. The (N)SPs were enzymatically isolated from the worts, and their physical characteristics were analyzed by asymmetric flow field‐flow fractionation coupled to multi‐angle light scattering and differential refractive index detectors (AF4‐MALS‐DRI; molar masses higher than 10 kDa) (Moreno Ravelo, Gastl, and Becker 2024a). Subsequently, the worts' lubrication was assessed with soft tribology to understand the impact of the grain source and the degree of modification in the FC response. This research demonstrates the presence of diverse (N)SPs structures, which influence the sample's FC (lubricity). The existence of polysaccharides with variant structures opens a new way to assess brewing research, especially on topics where shape can be important, such as filtration and sensory perception (e.g., *palate fullness* and *mouthfeel*).

## 2 Materials and Methods

### 2.1 Malting of Raw Grains and Their Standard Analysis

The raw grains used for the experiments were acquired from SAATEN UNION GmbH (Germany). The malting was performed on a micro‐malting system (1 kg scale) following MEBAK procedure R‐110.00.008 [2016‐03] for B grains (iso 65°C) (Jacob 2013). W and O grains followed the same procedure with slight variations as the germination times were 6 and 7 days, respectively. The grains were malted at two different levels by altering the SD at high (45%) and low (39%) generating six different malts.

Standard malt analysis following an iso‐thermal 65°C mash (R‐207.00.002) (Jacob 2013), according to MEBAK, was implemented to confirm the impact of the different SD levels on the malts: extract (R‐205.01.080 [2016–03]), viscosity (R‐205.10.282 [2016–03]), BGs content (R‐205.15.174 [2016–03]), soluble nitrogen (R‐205.11.030 [2016–03]), free‐amino nitrogen (R‐205.14.111 [2016–03]), final degree of attenuation (R‐205.17.080 [2016–03]), and friability (R‐200.14.011 [2016–03]).

### 2.2 Wort Production on Laboratory Scale

As the fermentation process does not alter the high molecular weight compounds in beer (Krebs, Becker, and Gastl 2020), boiled wort produced on a laboratory scale was employed to proof the presence of different molecular shapes of (N)SPs. The mashing procedure used was based on MEBAK's iso‐thermal 65°C (R‐207.00.002) (Jacob 2013). The following changes were performed to improve reproducibility: 50 g of milled malt (laboratory mill, LM 3100, Perten Instruments) was mixed with 350 mL of distilled water (63°C). An iso‐thermal mashing at 63°C for 2 h was used. After the first 30 min, 50 mL of distilled water (63°C) was added. Subsequent cooling down to room temperature followed to a weight adjustment to 450 g by adding distilled water. B and W samples were filtered by filter paper (514 ¼, diameter 320 mm, Macherey‐Nagel, Germany) and O samples by centrifugation (4000 rpm, 10 min, Heraeus MULTIFUGE 4KR 230 V 50HZ, DJBlabcare, England). At last, the worts were boiled for 25 min to later being hot filtered with filter paper and directly stored in metallic containers to prevent microbiological contamination. This procedure was performed on triplicate. The chemical analysis, as in the standard malt (iso‐65°C from section 2.1), was performed on all the iso‐63°C worts.

### 2.3 (Non‐)Starch Polysaccharides Isolation and Molecular Characterization of Worts

The methodology from (Moreno Ravelo, Gastl, and Becker 2024a) was used to extract and measure the molar mass and conformation of (N)SPs by AF4‐MALS‐DRI with minor modifications. 20 μL of endo‐1,4‐β‐xylanase (E‐XYLNP, Megazyme, Ireland) was used instead of (100 μL) E‐XYTR1. Before gel permeation chromatography fractionation, all samples were centrifuged three times (5000 rpm, 5 min., Sigma 6 k15 centrifuge, Sigma Laborzentrifugen GmbH, Germany) to prevent an excess amount of particles to enter and contaminate the column. Before their molar mass characterization, the samples were re‐dissolved in 4 mL bi‐distilled water and filtered with a 0.2 μm syringe filter (PES 20/25, Chromafil).

AF4 (Eclipse 3+, Wyatt Technology Europe, Germany) occurred on a long separation channel (tip‐to‐tip length of 26.5 cm, inlet width 2.1 cm, and outlet width 0.6 cm) with trapezoidal geometry with a 350 μm spacer. The actual channel thickness of the 10 kDa membranes (regenerated cellulose, Wyatt Technology Europe, Germany) was 256.8 ± 7.5 μm, calculated for BSA by using the FFFHydRad v. 2.0 MATLAB extension (Lund University, Sweden) as described by (Litzén 1993). An inline filter (0.1 μm) was placed between the quaternary pump (Agilent 1100 series, Agilent Technologies, Germany) and the autosampler (Agilent 1100 series, Agilent Technologies, Germany) to prevent noise in the detectors from big particles. The MALS detector used was DAWN HELEOS II from Wyatt Technology Europe (Germany) and the DRI‐detector from Agilent Technologies (Germany). The mass injected was adjusted to prevent channel overloading; the injection volume of B (N)SPs was 50 μL while 25 μL for the rest of grain samples. Elution conditions were a detector flow of 1 mL/min, initial cross‐flow of 3 mL/min with a half‐life of 4 min under an exponential decay (0.1 mL/min minimum cross‐flow). Samples were focused for 3 min under the same conditions as the initial cross‐flow.

The data was recorded and analyzed with ASTRA software (6.1.2 and 6.1.7 respectively, Wyatt Technology Europe, Germany). The data from 11 scattering angles (34.8°–142.5°) was fitted with the Berry formalism. A dn/dc of 0.146 was used, neglecting the second viral coefficient (A_2_). The conformation parameters ($r_{\mathrm{hyd}}$, $\rho_{\mathrm{app}}$) were calculated with FFFHydRad MATLAB extension as explained in detail somewhere else (Nilsson et al. 2006).

### 2.4 Tribology Determination of Worts

Wort lubrication was determined by soft tribology. The devices and consumables used to assess FC were acquired from Anton Paar GmbH (Germany). The T‐PTD 200 tribology was mounted on a MCR 502 WESP rheometer. A ball‐on‐3 pins configuration attachment with a steel ball (12.7 mm) and cylindrical PDMS (Sylgard 184) pins were chosen to measure the samples as they can mimic the palate and tongue properties, respectively (Biegler et al. 2016). The device was controlled and data was recorded with RheoCompass software (1.30.1227. Anton Paar GmbH) with the following settings: TruRate 80%, dynamic normal force 80%, and a maximum torque of 150 mN·m.

The sample holder was rinsed with distilled water, ethanol, and acetone followed by the addition of PDMS pins. After fixing the sample holder to the device, the motor adjustment and measurement of the system inertia were performed at a distance of 1 mm from the contact point. Subsequently, 500 μL of sample at room temperature was added. Wort samples were centrifuged (5000 rpm, 5 min) before their analysis to avoid any solid particle remaining in the liquid to intervene in the measurement. The measuring temperature was 27°C as this was reported to be the temperature in the mouth when consuming semi‐solid food at low temperature (Engelen et al. 2003). The steel sphere was lowered followed by an equilibration step where the measuring normal force of 6 N was achieved. The FC was assessed by logarithmically ramp‐down speed starting from 1 to 10^−8^ m/s. Each measurement was composed of three runs, the first and second runs were used to equilibrate the PDMS pins and reduce variability (running‐in) and only the third measurement was considered for calculations; a fresh aliquot was used for each run. The PDMS pins were exchanged after each sample measurement (3 runs). All samples were measured in duplicate.

### 2.5 Statistical Evaluation

OriginPro 2020 ver. 9.7.0.188 (OriginLab Corporation, United States) was used to calculate the average and standard deviation of the AF4‐MALS‐DRI during the elution. The average multiple curves option based on the same X (retention time) was used, which also reduced the 1081 recorded points to 100 points while keeping the data integrity. Principal component analysis (PCA) and partial least squares regression (PLSR) were performed in JMP Pro ver. 17.0.0 (SAS, Belgium). Calculations of conformation based on AF4‐MALS‐DRI were done with FFFHydRad ver. 2.0 extension in MATLAB R2022b.

## 3 Results and Discussion

The first part of this work is aiming to confirm the presence of different molecular conformations of (N)SPs in cereal‐based beverages. To accomplish this, grains from different sources were used because the (N)SPs' ratios (Havrlentova and Kraic 2006; Zannini et al. 2022), and molar masses naturally vary (Sun et al. 2021). Furthermore, the grains were malted at two SD levels to accentuate the chemical and molar mass variation as previously reported (Moreno Ravelo, Gastl, and Becker 2024b). The standard malt analysis (iso 65°C) confirmed that O samples contained the highest BGs content followed by B and W grains (Table S1).

### 3.1 Molar Mass and Conformation of (N)SPs From Laboratory‐Produced Worts

The malted grains were used to produce wort with an iso‐thermal temperature of 63°C. The chemical characteristics of the produced worts are presented in the Table S2. As expected, worts produced with O showed the highest BGs and lowest AXs concentrations. The AXs content of W worts was slightly higher than B worts, while their BGs content was the lowest. Thus, as expected, it can be confirmed that using malt from different grain sources effectively varied the chemical ratios of (N)SPs in the worts.

The (N)SPs were extracted, and their molar mass analysis was done using AF4‐MALS‐DRI. In turn, the conformation parameters were calculated based on their retention time following a numerical approach based on the system configuration. Different physical characteristics were observed on the (N)SPs (see Table 1). Each polysaccharide is independently discussed, showing the average value per grain used during the text discussion. AXs in O worts showed the highest $M_{w}$ (2140.3 kDa), followed by W (142.6 kDa) and B (125.65 kDa) worts. However, the biggest $r_{\mathrm{hyd}}$ was observed in W samples (12.4 nm), while B contained the smallest values (8.65 nm). The molar mass of AXs, calculated by size exclusion chromatography (SEC), has been reported in the literature for different grain sources, ranging from 10^4^ to 10^5^ g/mol (10–100 kDa, (Dervilly et al. 2002; Zhou et al. 2010)). Thus, the values obtained in this work for B and W grains are in a similar range. The $M_{w}$ found on O samples is slightly higher than literature values (up to 2000 kDa, (Tian, Gruppen, and Schols 2015)). This difference might be attributed to the different extraction methods used, as they highly influence the physical characteristics of AXs (Wang et al. 2020; Yilmaz‐Turan et al. 2020).

**TABLE 1:** Average and standard deviation values of molar mass and conformation of (N)SPs extracted from laboratory‐produced worts using different malt grain sources (*n* = 3). Different letters represent statistical differences by pairwise comparison (Tukey–Kramer HSD).

		Unit	B39	B45	O39	O45	W39	W45
Arabinoxylans	$M_{w}$	[kDa]	129.87 ± 7.08 c	121.42 ± 0.19 c	2700.33 ± 359 a	1580.26 ± 348.98 b	146.02 ± 15.61 c	139.18 ± 0.48 c
	PDI	[−]	1.6 ± 0 c	1.5 ± 0 c	29.8 ± 3.7 a	18.1 ± 4 b	2 ± 0.2 c	2 ± 0 c
	$r_{\mathrm{rms}}$	[nm]	8.4 ± 1.2 bc	7.4 ± 0.1 c	9.2 ± 0.3 abc	8 ± 0.5 bc	10.3 ± 1.8 ab	11.5 ± 0.8 a
	$r_{\mathrm{hyd}}$	[nm]	8.2 ± 0.8 d	9.1 ± 0.3 cd	10.7 ± 0.1 bc	10.2 ± 0.5 bc	12.8 ± 1.4 a	11.9 ± 0.4 ab
	$\rho_{\mathrm{app}\;\mathrm{hyd}}$	[kg/m^3^]	344.3 ± 41.1 ab	310.4 ± 22.4 b	396.7 ± 20.5 a	369.3 ± 11.4 ab	202.7 ± 13.9 c	229.9 ± 7.9 c
	$r_{\mathrm{rms}}/r_{\mathrm{hyd}}$	[−]	0.99 ± 0.16 a	0.81 ± 0.02 a	0.96 ± 0.02 a	0.93 ± 0.09 a	0.85 ± 0.1 a	1.18 ± 0.22 a
	Mass detected	[μg]	116.1 ± 4.7 c	132.6 ± 4.8 c	176.3 ± 4 b	175.4 ± 8.7 b	204.2 ± 8.1 a	211.5 ± 6 a
β‐glucans	$M_{w}$	[kDa]	126.06 ± 1.85 b	117.22 ± 3.35 b	20728.24 ± 29937.93	1677.3 ± 243.13 a	143.29 ± 2.03 b	142.21 ± 4.91 b
	PDI	[−]	1.5 ± 0 b	1.4 ± 0 b	11.6 ± 7.2	19.2 ± 2.3 a	1.8 ± 0.2 b	1.9 ± 0.1 b
	$r_{\mathrm{rms}}$	[nm]	7.9 ± 1.3 a	8 ± 0.9 a	136.5 ± 125.6	7.8 ± 1 a	6.1 ± 1.1 a	6.3 ± 0.3 a
	$r_{\mathrm{hyd}}$	[nm]	7.2 ± 0.1 b	7.4 ± 0.3 b	8.7 ± 0.1	9.1 ± 0.3 a	5.9 ± 0.3 c	6 ± 0.1 c
	$\rho_{\mathrm{app}\;\mathrm{hyd}}$	[kg/m^3^]	380.4 ± 10.2 a	380.2 ± 8.7 a	58779.8 ± 97055.5	379.6 ± 17.3 a	450.2 ± 125.5 a	415.9 ± 14.9 a
	$r_{\mathrm{rms}}/r_{\mathrm{hyd}}$	[−]	1.11 ± 0.18 a	1.15 ± 0.14 a	11.42 ± 9.08	0.98 ± 0.12 a	1.37 ± 0.67 a	1.06 ± 0.07 a
	Mass detected	[μg]	115.6 ± 10.5 b	118 ± 7 b	167.8 ± 11.4	163.7 ± 10.2 a	125.5 ± 1.3 b	126.9 ± 3.3 b
Dextrins	$M_{w}$	[kDa]	202.46 ± 60.92 c	141.09 ± 0.93 c	4482.74 ± 664.89 a	3629.07 ± 106.03 b	228.56 ± 23.85 c	200.8 ± 4.79 c
	PDI	[−]	4 ± 1.3 c	3 ± 0.1 c	92.5 ± 10 a	74.2 ± 2.3 b	4.5 ± 0.3 c	4.4 ± 0.1 c
	$r_{\mathrm{rms}}$	[nm]	14.5 ± 4.4 a	10.1 ± 0.1 a	12.8 ± 1.3 a	10.3 ± 2.1 a	9.3 ± 0.7 a	9.7 ± 1.7 a
	$r_{\mathrm{hyd}}$	[nm]	10.8 ± 0.6 b	10.9 ± 0.4 b	14.6 ± 1.1 a	14.8 ± 0.5 a	6.8 ± 0.3 c	7.6 ± 0.3 c
	$\rho_{\mathrm{app}\;\mathrm{hyd}}$	[kg/m3]	203.4 ± 9.8 a	173.1 ± 4.3 b	195.3 ± 3.1 ab	183.7 ± 20.3 ab	185.3 ± 10.3 ab	206.2 ± 3.8 a
	$r_{\mathrm{rms}}/r_{\mathrm{hyd}}$	[−]	1.07 ± 0.44 a	0.91 ± 0.02 a	1.01 ± 0.11 a	0.86 ± 0.17 a	1.08 ± 0.3 a	1.28 ± 0.24 a
	Mass detected	[μg]	45 ± 1.6 c	48.9 ± 1.8 bc	85.4 ± 10.1 a	79.6 ± 2.3 a	52.7 ± 1.9 bc	58.8 ± 1.2 b
(N)SPs	AXs	[%]	42	44.3	41	41.9	53.4	53.2
	BGs	[%]	41.7	39.4	39.1	39.1	32.8	32
	DXs	[%]	16.3	16.3	19.9	19	13.8	14.8

Regarding the conformation parameters, AXs from O worts presented the highest $\rho_{\mathrm{app}\;\mathrm{hyd}}$ (383 kg/m^3^). The range of the conformation ratio, $r_{\mathrm{rms}}/r_{\mathrm{hyd}}$, of AXs in all the grains (0.81–1.18) indicates the presence of different polysaccharide shapes: micro‐gel structure ($r_{\mathrm{rms}}/r_{\mathrm{hyd}}$ = 0.5–1, (Zielke, Stradner, and Nilsson 2018)), sphere ($r_{\mathrm{rms}}/r_{\mathrm{hyd}}$ = 0.775), and hyper‐branched polysaccharides ($r_{\mathrm{rms}}/r_{\mathrm{hyd}}$ = 1.23). Although the average $r_{\mathrm{rms}}/r_{\mathrm{hyd}}$ values reported in Table 1 are reasonable estimates for dominant conformations, multiple conformations may be present at different molar masses within each (N)SPs. Therefore, observing the conformation behavior by plotting $r_{\mathrm{rms}}/r_{\mathrm{hyd}}$ and $\rho_{\mathrm{app}\;\mathrm{hyd}}$ along the molar masses is relevant. This information can be seen in the Figure S1.

AXs extracted from brewing grains have shown diverse structures. The Mark‐Houwink‐Sakurada constant (α) values reported in the literature suggests the presence of random coil (α = 0.65–0.75, (Shaluk et al. 2019)) and rod AXs molecules (α = 1–2, (O Marconi et al. 2020)) while the $r_{\mathrm{rms}}/r_{\mathrm{hyd}}$ found similar structures compared to this work (0.7–1.2) corresponding to micro‐gel, sphere, and hyper‐branched molecules (Moreno Ravelo, Gastl, and Becker 2024a). A possible explanation for these differences could be attributed to the different methods used for molar mass fractionation, as SEC, which is commonly used to measure α from the Mark‐Houwink‐Sakurada equation, may lead to shear degradation on the molecules (Podzimek 2022; Rolland‐Sabate et al. 2011). Despite their average $r_{\mathrm{rms}}/r_{\mathrm{hyd}}$ values indicate a major presence of different structures (Table 1), AXs presented random coil and rod structures in the samples at low molar masses (< 10^5^ g/mol, Figure S1).

Different effects were observed when analyzing the impact of the modification level. A higher SD led to a decrease in molar mass and conformation changes of AXs in B and O. Their structures become more compact upon higher SD, which is observed by their decreased in $r_{\mathrm{rms}}/r_{\mathrm{hyd}}$ (B39 0.99 → B45 0.81; O39 0.96 → O45 0.93) and $\rho_{\mathrm{app}\;\mathrm{hyd}}$ (B39 334.3 kg/m^3^ → B45 310.4 kg/m^3^; O39 396.7 kg/m^3^ → O45 369.3 kg/m^3^). Despite the molar mass of AXs presents a similar behavior to the other grains, AXs from W malt showed a different conformation behavior. The $\rho_{\mathrm{app}\;\mathrm{hyd}}$ and $r_{\mathrm{rms}}/r_{\mathrm{hyd}}$ values increased when a high SD was used (e.g., W39 0.85 → W45 1.18), hinting at the presence of branched or aggregated structures. Based on the conformation behavior at different molar masses (Figure S1), the density decrease upon molar mass increase rejects the hypothesis of the presence of aggregates in AXs in W45. Thus, the existence of branched AXs in W45 is suggested. Branched AXs have been previously described in highly modified B malt (Shaluk et al. 2019).

Contrasting results regarding the molar mass and conformation changes of AXs in malt are found in the literature. While some authors agreed with our results for the molar mass decrease in W malt, attributed to a higher enzymatic activity due to further malting modification by means of SD or germination temperature (Dervilly et al. 2002; Guo et al. 2014), others found a molar mass increase (Shaluk et al. 2019). The latter research concluded that the genotype of the grain source should impact these differences. Marconi et al. later confirmed this by observing a molar mass variation of AXs according to the (B) genotype and harvest year (Marconi et al. 2020). According to Shaluk et al., no clear relationships have been found between conformation and malting degree (SD) in two B malt varieties (Shaluk et al. 2019). To the knowledge of the authors, no information on W and O grains is available on this topic.

Regarding BGs, the $M_{w}$ was the highest on the O sample (only O45 was considered), followed by W (142.75 kDa) and B (121.64 kDa). The $\rho_{\mathrm{app}\;\mathrm{hyd}}$ was higher in W (433.05 kg/m^3^), while it was comparable in B (380.3 kg/m^3^) and O (379.6 kg/m^3^) samples. Regarding the $r_{\mathrm{rms}}/r_{\mathrm{hyd}}$, O samples presented a more compact, close to a sphere‐like structure (O45 0.98) compared to the more elongated structures found in B (1.13) and W (1.22). Similar conformation values have been observed in BGs extracted from raw O and B grains analyzed by AF4‐MALS‐DRI, but higher molar masses were reported (Zielke, Stradner, and Nilsson 2018). This difference can be attributed to the malting process as it degrades the BGs, hence decreasing its molar mass. Nevertheless, other research has found comparable molar masses in barley malt as this work (Marconi et al. 2014), hence validating our reported values.

The $M_{w}$ of BGs decreased within B and W samples upon a higher SD, but the changes were not significant. These results are not surprising as BGs are well known to be mainly degraded during malting, affecting their molar mass (O. Marconi et al. 2014). A more pronounced variation was observed in the conformation parameters. $\rho_{\mathrm{app}\;\mathrm{hyd}}$ remained constant in B worts (B39 380.4 kg/m^3^ → B45 380.2 kg/m^3^) and it decreased on W samples (W39 450.2 kg/m^3^ → W45 415.9 kg/m^3^). The decrease in $r_{\mathrm{rms}}/r_{\mathrm{hyd}}$ on BGs in W samples indicates that the higher SD generates more compact polysaccharides (W39 1.37 → W45 1.06), while the slight increase in B samples (B39 1.11 → B45 1.15) suggests hyper‐branched structures. However, the latter is not possible because BGs from cereals are linear polysaccharides (Izydorczyk and Dexter 2008). When analyzing the conformation behavior plots on the molar mass range where the $r_{\mathrm{rms}}/r_{\mathrm{hyd}}$ suggests a hyper‐branched structure (Figure S1), the $\rho_{\mathrm{app}\;\mathrm{hyd}}$ decreases in all the grains, hinting the presence of individual aggregates. This behavior has previously been reported in the literature for NSPs (Dervilly et al. 2002; Pitkanen, Tenkanen, and Tuomainen 2011). Furthermore, similar results are available in the literature regarding conformation changes upon malting in B malt: a more compact conformation (α constant decrease, (Marconi et al. 2014)) and a slightly more elongated structure (α constant increase, (Shaluk et al. 2019)). The enzymatic digestion of BGs during malting might explain these changes. The O39 sample showed a high standard deviation, in which the co‐elution of big particles in the separation channel might be responsible. These particles cannot be retained in the separation channel, thus changing their elution time earlier than expected (steric mode of FFF). The size of the peak apex of these big particles is 250 nm, which is close to the steric transition point of 0.3–3 μm (Podzimek 2011). Thus, it can be suggested that their big size impeded their correct fractionation and, hence, physical characterization. Due to the absence of these big particles in the O45 sample, their degradation during malting can be suggested.

DXs from O presented the highest $M_{w}$ (4055.91 kDa), polydispersity (PDI), and size. Their $r_{\mathrm{rms}}/r_{\mathrm{hyd}}$ suggests a compact sphere‐like structure (0.94). B's DXs showed the lowest $M_{w}$ (171.78 kDa) with similar conformations to O. The DXs in W samples showed a middle $M_{w}$ response (214.68 kDa), but their size was the smallest. The average $r_{\mathrm{rms}}/r_{\mathrm{hyd}}$ indicates the presence of branches ($r_{\mathrm{rms}}/r_{\mathrm{hyd}}$ = 1–1.5, (Zielke, Stradner, and Nilsson 2018)) on both W worts (1.18), showing the highest densities among the grains (195.6 kg/m^3^).

The higher SD during malting decreased the DXs response on most of the parameters on B and O (generation of compact structures). However, an opposite behavior was observed in W samples where the conformation parameters increased. Furthermore, W45 (1.28 ± 0.24) contains more branched DXs than W39 (1.08 ± 0.3). These branches can be confirmed when comparing the $\rho_{\mathrm{app}\;\mathrm{hyd}}$, which is higher on W45. Nevertheless, these branches were also present in B39 and O39 samples due to the higher $r_{\mathrm{rms}}/r_{\mathrm{hyd}}$ and $\rho_{\mathrm{app}\;\mathrm{hyd}}$ compared to B45 and O45, respectively.

The DXs' molar masses assessed by chromatography techniques are usually lower than the values in Table 1 (Enevoldsen and Schmidt 1974). However, a similar order of magnitude was found in our previous work (Moreno Ravelo, Gastl, and Becker 2024a). Since DXs are products of hydrolyzed starch, gelatinization temperature and enzymatic content (diastatic power) may influence their physical characteristics. In addition, the intrinsic starch characteristics, such as molar mass and amylose to amylopectin ratio, are also relevant. For example, starch from O presented a higher molar mass (5.4·10^7^ g/mol) compared to B (4.3·10^7^ g/mol) under the same extraction and measurement conditions (Kurdziel et al. 2019). Additionally, these grains may contain a different amylose content (Bertoft 2017), which ratio to amylopectin can influence molar mass responses (You and Izydorczyk 2002).

Despite the malting process generates amylolytic enzymes necessary for the brewing process, the main saccharification occurs during the mashing step (Back et al. 2019; Langenaeken et al. 2020a). The enzymatic digestion during mashing depends on the gelatinization temperature (Langenaeken et al. 2019). Nevertheless, this should not influence the observed DXs´ molar mass differences among the different grains because their gelatinization temperature is similar. Thus, it can be assumed that the DXs' molar mass reduction upon further malt modification, observed in all the grains at higher SD, could be explained by a higher content of amylolytic enzymes (Surmiński, Masior, and Kuchciak 1986). Similarly, the higher amount of branched DXs, hinted by a higher $r_{\mathrm{rms}}/r_{\mathrm{hyd}}$ on low SD grains, can be attributed to a lower amylolytic power, specifically to a lower β‐amylase and limit dextrinase activity. The DXs conformation can be present as linear and branched polysaccharides according to the literature (Enevoldsen and Schmidt 1974; Moreno Ravelo, Gastl, and Becker 2024a), thus affirming the wide variety of structures hinted at the calculated $r_{\mathrm{rms}}/r_{\mathrm{hyd}}$ values.

The proportion of extracted (N)SPs, based on the mass detected per treated malt grain by AF4, was also calculated and displayed in Table 1. More than half the amount of (N)SPs detected in W grain worts was AXs, followed by BGs and DXs. The same order was observed on the B and O samples, but the order of magnitude changed. The main difference between B and O samples was the DXs proportion, being steadier on B samples.

**FIGURE 1:**
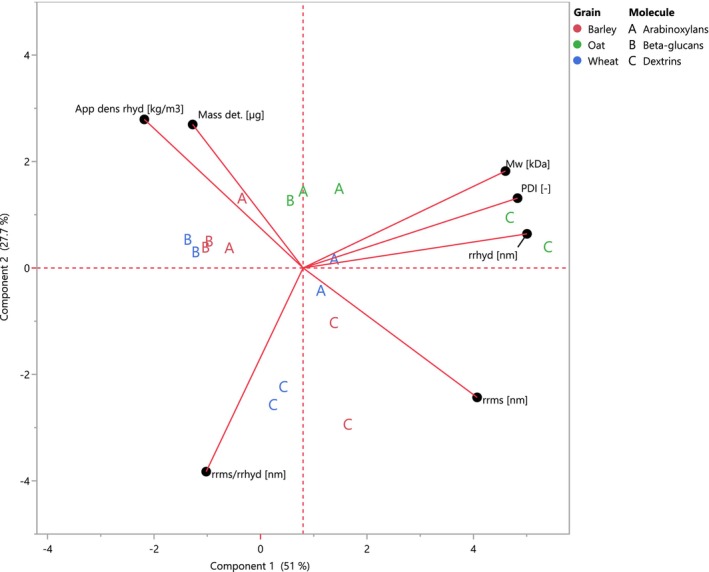
PCA of average molar mass and conformation of (N)SPs extracted from laboratory‐produced worts using different malt grain sources (*n* = 3). Each letter represents different (N)SPs: A. AXs; B. BGs; C. DXs (For interpretation of the references to color in this figure legend, the reader is referred to the web version of this article).

PCA was performed for better data interpretation. The resulting bi‐plot is shown in Figure 1. The data from O39 was not considered for the analysis. The first two principal components (PCs) explain 78.7% of the data variation. Based on their score position, AXs and DXs showed more diversity in their physical characteristics among the malted grains. DXs from O have the highest molar mass, PDI, and $r_{\mathrm{hyd}}$. Meanwhile, DXs from W were characterized for presenting highest the $r_{\mathrm{rms}}/r_{\mathrm{hyd}}$.

BGs from B and W showed similar characteristics: low molar mass, PDI, and size, being $\rho_{\mathrm{app}\;\mathrm{hyd}}$ higher in W samples. They also presented a more elongated structure ($r_{\mathrm{rms}}/r_{\mathrm{hyd}}$ > 1) than O45, which showed a higher PDI and molar mass than the previously mentioned BGs from B and W samples. At last, AXs on W presented the bigger size ($r_{\mathrm{hyd}}$), while a higher density was predominant on B samples. Similar to BGs and DXs, the molar mass and PDI on AXs in O were higher than the other grains.

### 3.2 Soft Tribology of Laboratory‐Produced Worts

**FIGURE 2:**
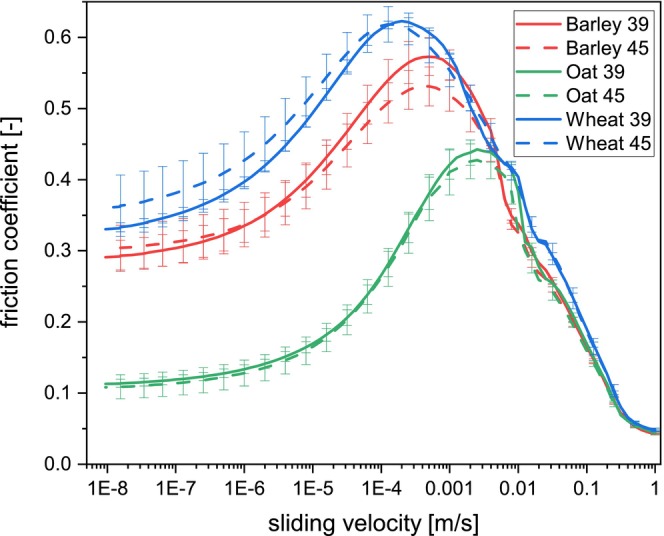
Stribeck curves of wort samples (iso 63°C, *n* = 3) produced with malt from different sources (barley red, oat green, wheat blue) modified at two different levels with SD as parameter (full line 39%, dash line 45%). The error bar represents the standard deviation.(For interpretation of the references to color in this figure legend, the reader is referred to the web version of this article).

The FC was measured on the worts produced from different grain sources modified at two levels. The results are displayed as Stribeck curves (Figure 2). A wide sliding velocity profile was used to analyze all the lubrication regimes. Nevertheless, the elastohydrodynamic regime was not present. This regime is dominated by fluid dynamics (viscosity) (Stokes, Boehm, and Baier 2013), suggesting that the difference in viscosity among the samples is slight to influence the presence of the elastohydrodynamic regime under the current conditions. This absence was also observed for various non‐alcoholic beers (Fox et al. 2022). Furthermore, the surface hydrophobicity, as in PDMS pins, delays the appearance of the elastohydrodynamic regime by preventing full film lubrication (Stokes 2012). Although the elastohydrodynamic regime was absent, its transition to the mixed regime was present at high velocities (1 m/s).

When a ramp‐down velocity is applied, three lubrication regimes were observed on wort samples: static, boundary, and mixed. Consequently, each regime represents different film thicknesses between the surfaces (steel ball and PDMS pin). As the gap between the surfaces decreases due to a decrease in sliding velocity, the mixed regime arises while the FC increases. Despite a similar behavior in the mixed lubrication regime, the transition from the mixed to boundary regime varied depending on the grain source (FC apex). O samples required a sliding velocity of around 3E^−3^ m/s to reach the transition point, changing to 5E^−4^ m/s and 1E^−4^ for B and W, respectively. The boundary regime was observed after reaching the peak maximum upon further sliding velocity decrease. However, a clear “breakaway point,” as reported on extended Stribeck curves on food samples (Pondicherry, Rummel, and Laeuger 2018), was not present, hindering the transition between the boundary and static regimes ordinarily present at low sliding velocities. The reason behind this might be related to the implementation of the ramp‐down velocity profile. This “breakaway point” represents the starting of macroscopic motion (Pondicherry, Rummel, and Laeuger 2018), which is already occurring when a ramp‐down velocity is implemented.

**FIGURE 3:**
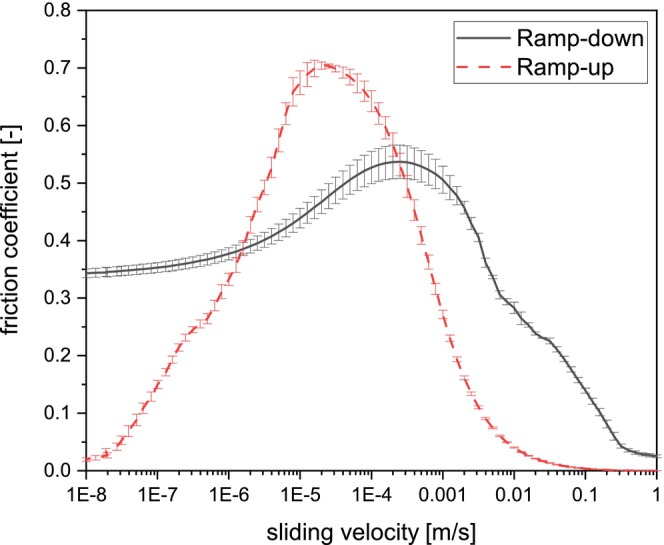
Stribeck curves of non‐alcoholic beer (< 0.5% ethanol) measured with different ramping velocities. The error bars represent the standard deviation of triplicate measurements (*n* = 3) (For interpretation of the references to color in this figure legend, the reader is referred to the web version of this article).

Precaution is needed when comparing tribology data because tribology is a system property (Fox et al. 2020). Our data agrees with the previously mentioned lack of an elastohydrodynamic regime and a similar order of magnitude for FC compared to published data of cereal‐based beverages (Fox et al. 2022). However, the Stribeck curve showed different behaviors. Regardless of employing similar instrument configuration (ball‐on‐3‐pins attachment), both works used different surfaces, velocity ramps, and sample handling. Hydrophobicity differences from using different (upper) surfaces could explain the disparity in Stribeck curve shapes (Bongaerts, Fourtouni, and Stokes 2007). However, a similar curve shape was obtained when a bottom‐fermented non‐alcoholic beer was measured using the same procedure as the previously reported work, where even the “breakaway point” between the static and boundary regimes was shown (4.5·E^−7^ m/s, Figure 3). Thus, the distinct Stribeck curve behavior between our work and previous research (Fox et al. 2022) is due to employing different ramp velocities. Furthermore, the difference in sample handling might also emphasize these differences (Kieserling, Schalow, and Drusch 2018). Although the ramp velocity is essential (Fox et al. 2020), more research needs to be performed to understand how it influences the FC and lubrication regimes on food, especially cereal‐based beverages.

The grain source influenced the FC responses. The W samples showed the highest FC on both mixed and boundary regimes, while the lowest values were observed on O samples at the boundary/static regimes. This suggests that O worts bear the highest lubricity. Nevertheless, the differences are evident at the maximum FC point (the transition between boundary and mixed regimes). This point was also relevant when analyzing the malting effect (t‐student, letter in parenthesis); W39 showed a similar maximum FC (0.624 ± 0.007^A^) compared to W45 (0.620 ± 0.025^A^). B39 (0.574 ± 0.028^B^) was statistically lower than both W samples but statistically higher than B45 (0.532 ± 0.027^C^). As previously mentioned, O39 (0.443 ± 0.016^D^) and O45 (0.429 ± 0.024^D^) were statistically the samples with the lowest maximum FC. Although only B showed a significant difference due to the SD, the maximum FC was always lower within the same grain source on grains treated at higher SD.

The previously described maximum FC may appear arbitrary but has physiological relevance regarding oral processing. This point represents the transition between boundary and mixed regimes, which are essential regimes for food oral processing (Selway and Stokes 2014; Steinbach et al. 2014). Furthermore, the tongue's velocity recorded in the mouth while drinking water (3–5 mL) is in the range of 0.003–0.305 m/s, with a registered velocity decrease when the tongue is in contact with the palate (Peng et al. 2000). These values significantly differ depending on the volume to be swallowed, recording higher velocities with a higher volume, whereas the pressure remained similar (Kodama et al. 2021). Due to the sliding velocities at the maximum FC are in a similar range (especially in O samples) compared to the mouth, the maximum FC may also function as a relevant parameter for (drinking) oral processing and, hence, predicting human sensory perception.

### 3.3 Relationship Between Wort's Chemical and Physical Characteristics and Their Friction

**FIGURE 4:**
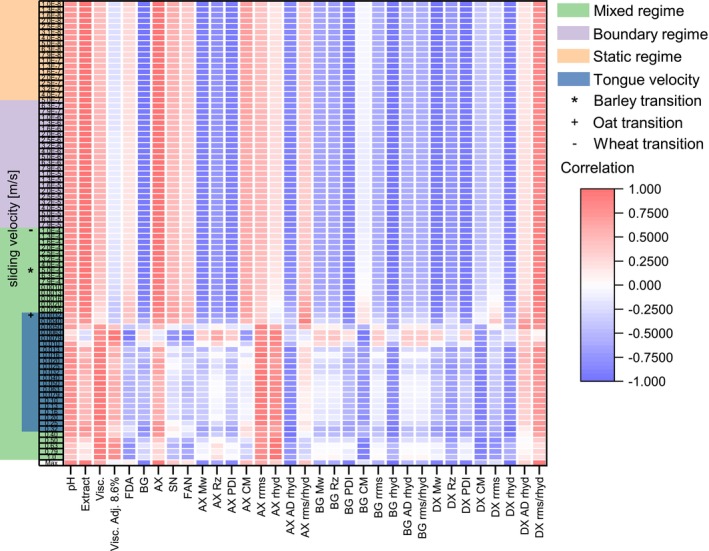
Correlation heat map of FC at different sliding velocities (m/s) with average chemical and physical wort responses. The tongue velocity values were taken from (Peng et al. 2000) (For interpretation of the references to color in this figure legend, the reader is referred to the web version of this article).

The effect of the multiple parameters measured to FC at different sliding velocities was analyzed using a correlation analysis based on the average responses on all the laboratory‐produced worts (Figure 4). The transition point for the static regime was chosen based on the ramp‐up velocities results from Figure 3. Minor differences in the direction and the magnitude of the correlation can be observed in the FC values in the boundary and static regimes of all the measured parameters. Changes in correlation direction and higher magnitudes were observed in the velocity range of tongue movement (Peng et al. 2000), which is part of the mixed regime. Most of the parameters displayed the same correlation direction at all the sliding velocities except adjusted viscosity 8.6%, final degree of attenuation, soluble nitrogen, free‐amino nitrogen, AXs´ calculated concentration, and BGs´ calculated concentration that presented a change at 0.0040 m/s.

The correlation analysis of maximum FC to wort chemical analysis, molar mass, and conformation of (N)SPs shows that different factors may influence the FC response. The molar mass of AXs (−0.848, *p* < 0.05), BGs (−0.876), and DXs (−0.897, *p* < 0.05) show high correlation values to this parameter. The concentration measured in wort of the BGs (−0.883, *p* < 0.05) and AXs (0.926, *p* < 0.05) present significant correlations to maximum FC. The final degree of attenuation, which could be interpreted as an indirect way to measure DXs (Moreno Ravelo, Gastl, and Becker 2024b), did not show any correlation. Other parameters such as PDI of all (N)SPs[−], $\rho_{\mathrm{app}\;\mathrm{hyd}}$ of AXs[−], $r_{\mathrm{hyd}}$ of BGs[−] and DXs[−], $r_{\mathrm{rms}}/r_{\mathrm{hyd}}$ of DXs[+], and wort extract[+] also showed significant correlations to FC, with an absolute value of 0.850 as the minimum found (sign in brackets represents the correlation direction).

No data regarding correlations between tribology and chemical analysis of wort has been reported in the literature. However, the negative correlations of the (N)SPs' concentration detected behave according to the literature as higher concentrations of polysaccharides are reported to lower FC values in the boundary and mixed regimes (de Vicente, Stokes, and Spikes 2006). Furthermore, the conformation of polysaccharides was reported as relevant for tribology (You and Sarkar 2020), which also shows a positive correlation to DXs´ conformation ($r_{\mathrm{rms}}/r_{\mathrm{hyd}}$). It is essential to point out that the tribology measurements were performed on the whole wort samples, so the FC values result from all the analytes present (e.g., proteins, polyphenols, sugars). Thus, further research with a more extensive data set would be needed to confirm the individual parameters influencing FC in wort samples.

Polysaccharides are polydispersed systems, making determining their relevance with a single averaged parameter difficult, as shown in Figure 4. To improve this, the physical characteristics of the (N)SPs during the whole elution time in the AF4 separation channel were used. This analysis allowed to determine the main characteristics of the (N)SPs influencing the FC response. PLSR was used to determine each parameter's importance and direction. Before the PLSR, PCA on each AF4 parameter was independently performed to reduce the data dimensionality. Only the PCs with an eigenvalue higher than one were used as independent variables (data not shown). The dependent variables were the FC at multiple sliding velocities (16 points based on the correlation behavior of Figure 4).

**FIGURE 5:**
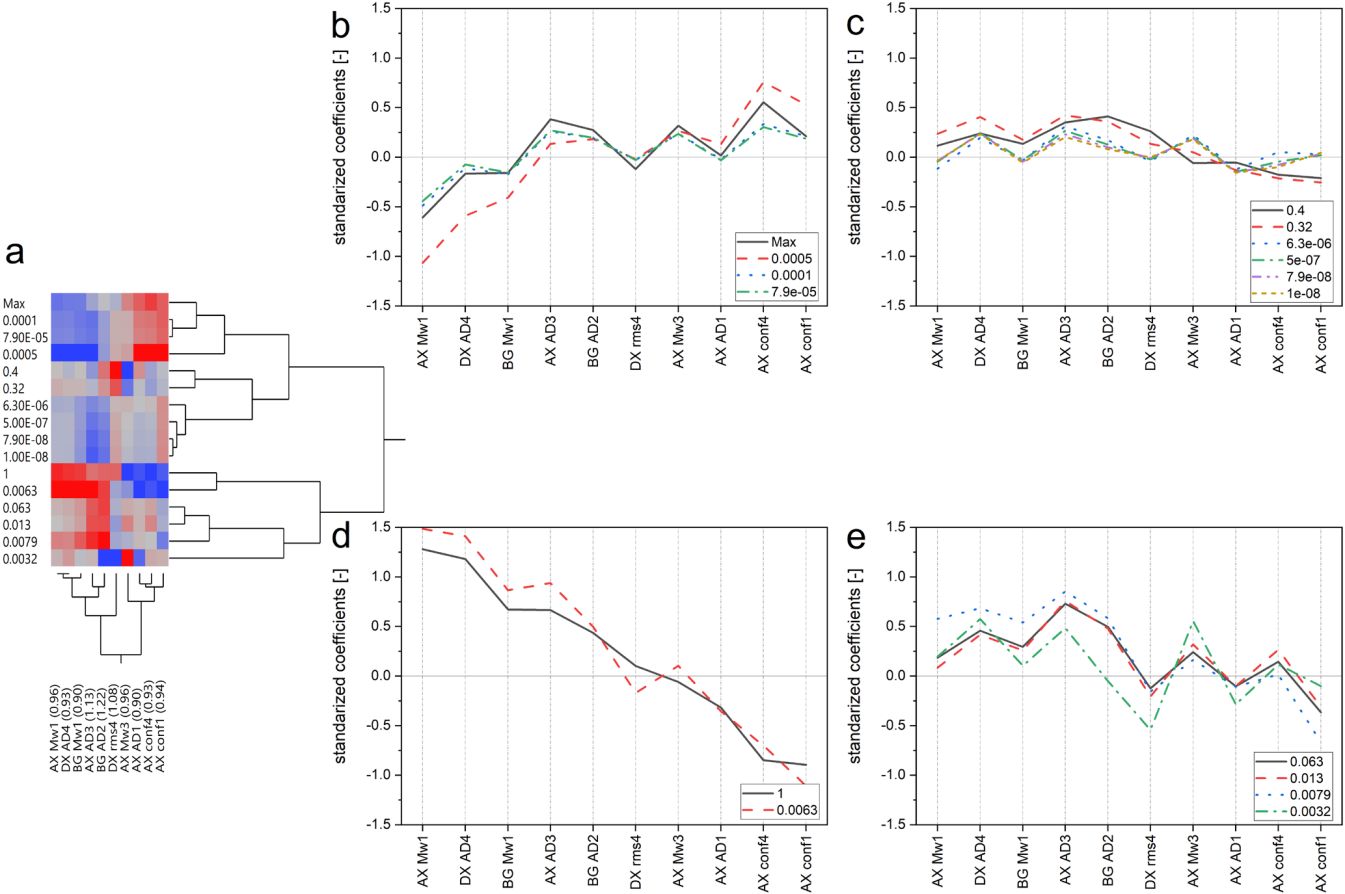
Two‐way hierarchical clustering of standardized PLSR coefficients (a) performed with parameters with a VIP > 1.3. The red and blue color depicts a higher and lower standardized value, respectively. The clustered data is also shown in line charts (b–e). The number of the principal component (PC) can be observed after each physical parameter. AD, ρapp hyd; AX, arabinoxylans; BG, β‐glucans; conf, Rrms/rhyd; DX, dextrins. (For interpretation of the references to color in this figure legend, the reader is referred to the web version of this article).

The first PLSR showed that only two factors were necessary to explain the model (data not shown). From this analysis, only the PCs showing a variable of importance (VIP) score of 1.3 were used for a second PLSR. The standardized coefficients resulting from the second PLSR are shown in Figure 5. According to this information, the molar mass of AXs and BGs, the apparent density of all (N)SPs, the size of DXs, and the conformation of AXs influence the FC depending on the sliding velocity used. A two‐way hierarchical clustering was used on the standardized coefficients to ease the data interpretation, forming four clusters (Figure 5a). Cluster 1 (Figure 5b) was composed of sliding velocities from the mixed and boundary regimes, cluster 2 (Figure 5c) of a combination of all sliding velocities, and cluster 3 (Figure 5d) and 4 (Figure 5e) of velocities of the boundary regime.

Cluster 4 only grouped the sliding velocities found in the mouth: 0.013, 0.063, 0.0032, and 0.0079 m/s (Peng et al. 2000). The other sliding velocities of 0.32 and 0.0063 m/s, which can also be found in the mouth, were part of clusters two and three, respectively. Apart from 0.0063 m/s, a similar standardized coefficient behavior was observed on all the sliding velocities analog to the mouth (Figure 5c,e). Therefore, the same parameters broadly explain the FC within the in‐mouth velocity perspective, being the PCs of DX $\rho_{\mathrm{app}\;\mathrm{hyd}}4$, AX $\rho_{\mathrm{app}\;\mathrm{hyd}}3$, BG $\rho_{\mathrm{app}\;\mathrm{hyd}}2$, AX $M_{w}1$, AX $M_{w}3$, and BG $M_{w}1$ positively relevant. On the other hand, the mouth velocity with a different behavior (0.0063 m/s) showed a positive response to the PCs of AX $M_{w}1$ and DX $\rho_{\mathrm{app}\;\mathrm{hyd}}4$, while the maximum FC contained in cluster 1 is mainly influenced by AX $r_{\mathrm{rms}}/r_{\mathrm{hyd}}4$.

Because this analysis was based on data reduced by PCA, the loadings from each parameter positively influencing the FC can be used to interpret the relevant fractions based on the AF4's retention time (Figure S2). The summary of the physical characteristics' range among the samples (except O39) and their relationship to the sliding velocity is observed in Table 2. The relevant molar mass range of AXs is 46.75 to 82,600 kDa for the sliding velocities in cluster 3. Only a minor range of these molar masses positively influences the velocities of clusters 2 and 4 (41.94–80.88 kDa), relevant from an in‐mouth perspective. On the other hand, the molar mass of BGs of 70.4 to 416,000 kDa influences the sliding velocities in clusters 3 and 4.

**TABLE 2:** Physical characteristics of (N)SPs that are relevant depending on the tribology´ sliding velocity. The retention time (RT) for each PC was selected based on its loading values (Figure S2). The minimum and maximum values within each RT were extracted from all wort samples based on their RT with the exception of O39.

PCs	Unit	Sliding velocity relationship [m/s]	RT [min]	Minimum	Maximum
AX $M_{w}1$	[kDa]	Cluster 3	> 3.5 min	46.75	82,600
AX $M_{w}3$	[kDa]	Cluster 2 and 4	2.4–3	41.94	80.88
AX $\rho_{\mathrm{app}\;\mathrm{hyd}}3$	[kg/m^3^]	Cluster 2, 3, and 4	2.4–3.9	169.35	935.37
			11–19.7	0.63	56.93
AX $r_{\mathrm{rms}}/r_{\mathrm{hyd}}4$	[−]	Cluster 1	5–7.5	0.89	1.58
DX $\rho_{\mathrm{app}\;\mathrm{hyd}}4$	[kg/m^3^]	Cluster 3	2.4–3	216.52	552.94
			6.5–9.7	9.06	37.58
BG $M_{w}1$	[kDa]	Cluster 3 and 4	3.5–6	70.4	113
			8–15.75	110	34,600
			17.5–19.7	1940	416,000
BG $\rho_{\mathrm{app}\;\mathrm{hyd}}2$	[kg/m^3^]	Cluster 2 and 4	2.4–6	40	991.16
			13–17	1.38	173.17
			18.2–19	0.74	179.09

The conformation of early eluting (N)SPs (2.4–6 min retention time) was relevant at different sliding velocities. Here, the polysaccharides present the highest density values. Based on the conformation ratio values obtained from AX $r_{\mathrm{rms}}/r_{\mathrm{hyd}}4$, the sphere, branched, and random coiled AXs structures are relevant for the sliding velocities of cluster 1. Although these parameters have not been investigated in wort/beer, the literature has reported the relevance of molar mass and structure of polysaccharides in other foods (Ji et al. 2023; Kieserling et al. 2019). Hence, the physical characteristics of (N)SPs positively contribute to the FC responses in laboratory‐produced worts.

## 4 Conclusions

This work confirms the presence of a broad spectrum of (N)SPs in laboratory‐produced wort using AF4‐MALS‐DRI. The grain source and its modification during the malting process by different SD levels significantly impacted the resulting molar mass and conformation. Due to their polydisperse nature, average values were used to analyze the samples´ molar mass and conformation differences. Although this approach analyzes the most predominant structures, the existence of the analyzed structures was verified by plotting the state of $r_{\mathrm{rms}}/r_{\mathrm{hyd}}$ upon molar mass change. The high SD produced lower molar masses on all the analyzed (N)SPs. O grains showed higher molar masses compared to W and B grains. The $r_{\mathrm{rms}}/r_{\mathrm{hyd}}$ found in NSP (AXs and BGs) indicates the presence of micro‐gel (0.5–1) and sphere (0.775) structures, while hyper‐branched (1.23) structures in addition to the previously mentioned were found in DXs and AXs. The effect of malting was observed differently in the (N)SPs depending on the grain source. In general, the higher modification decreased most of the measured parameters of BGs on all the grains, suggesting the formation of compact structures upon malt modification. The lack of enzymes produced in the low modification grains was attributed to a higher presence of branched DXs in all the grains ($r_{\mathrm{rms}}/r_{\mathrm{hyd}}$ ≈ 1.23). On the other hand, while most of the conformation parameters decreased or remained relatively similar upon further malt modification, the AXs and DXs structures and density increased, indicating a higher amount of branched polysaccharides.

The soft tribology results, depicted as Stribeck curves, showed the presence of static, boundary, and mixed lubrication regimes. The ramp‐down velocity did not allow the distinction between the static and boundary regimes. This effect was caused by the ramp‐down velocity profile used to measure the wort samples. Implementing different grain sources led to multiple friction responses, recording the lowest values in the O samples in the mixed and static/boundary regimes and suggesting better lubrication, followed by the B and W samples. Although not statistically significant, the FC was higher in the samples treated with a higher SD. The analysis of the averaged physical and chemical wort data and the FC at different sliding velocities demonstrated the high relevance of the velocity range related to the human mouth due to the correlations found. Due to the polydisperse nature of (N)SPs and the correlation behavior depending on the sliding velocity, a clustering of the standardized coefficients from a PLSR model was used to elucidate the specific physical characteristics relevant to FC. The molar mass, $\rho_{\mathrm{app}\;\mathrm{hyd}}$, and $r_{\mathrm{rms}}/r_{\mathrm{hyd}}$ of AXs contributed positively to the FC response. Similarly, the $\rho_{\mathrm{app}\;\mathrm{hyd}}$ of BGs and DXs and the molar mass of BGs are positively influencing the FC. When the mouth velocity is only considered, only the $r_{\mathrm{rms}}/r_{\mathrm{hyd}}$ of AXs is irrelevant. Thus, it can be concluded that the lubrication behavior is influenced by the physical characteristics of (N)SPs in wort. Further research is needed to elucidate the individual contribution of (N)SPs with various physical characteristics to their individual FC.

These results bring a new perspective into the brewing industry, especially for the malting process. Generating new raw materials derived from altering the SD during malting can incorporate (N)SPs with different physical characteristics into the brewing process. The relevance of these structures has been proposed in the literature as they may influence the sensory characteristics of the sample, especially the *palate fullness* and *mouthfeel* attributes. However, further experimentation is required to confirm the presence of these structures after the brewing process and how they affect the FC and human sensory response. Despite this, combining soft tribology and the physical characterization of (N)SPs is a promising strategy to further characterize beer/NABs.

## Author Contributions

**Rolando Cesar Moreno Ravelo:** conceptualization (lead), formal analysis (lead), investigation (lead), methodology (lead), project administration (lead), visualization (lead), writing – original draft (lead). **Martina Gastl:** conceptualization (supporting), writing – review and editing (equal). **Thomas Becker:** writing – review and editing (equal).

## Ethics Statement

Compliance with ethics requirements. All procedures performed in studies involving human participants were in accordance with the ethical standards of the institutional and national research committee and with the 1964 Helsinki declaration and its later amendments or comparable ethical standards.

## Conflicts of Interest

The authors declare no conflicts of interest.

## Supporting information


Data S1.

## Data Availability

The data that supports the findings of this study are available in the Supporting Information of this article. The raw data of this study are available from the corresponding author upon reasonable request.
