# Supplementary material for: Relationship Between Physical Characteristics of Cereal Polysaccharides and Soft Tribology—The Importance of Grain Source and Malting Modification
Source: Food Sci Nutr. 2025 Jan 7;13(1):e4699. doi: 10.1002/fsn3.4699 (PMC11717023; doi:10.1002/fsn3.4699)
Supplement: Supplementary file 1 — Data S1. [file FSN3-13-e4699-s001.docx]

**Supplementary material**

**Relationship between physical characteristics of cereal polysaccharides and soft tribology – The importance of grain source and malting modification**

Rolando César Moreno Ravelo*^1^, Martina Gastl^2^, Thomas Becker^1^

^1^Technical University of Munich, Institute of Brewing and Beverage Technology, Research

Group Raw Material Based Brewing and Beverage Technology, 85354, Freising, Germany

^2^Technical University Munich, Research Center Weihenstephan for Brewing and Food Quality.

Tel.: +49 8161 71-5170. Fax: +49 8161 71-4181

*Corresponding author. E-mail address: [rolando.moreno-ravelo@tum.de](mailto:rolando.moreno-ravelo@tum.de)

ORCID: Rolando César Moreno Ravelo 0000-0002-1278-870X

Table S1. Standard malt analysis of malt from different sources (iso 65 °C, n = 3)

| Sample | S.D. | pH-Wert | Extract | Viscosity | Color | β-Glucan | Soluble Nitrogen | FAN | FDA | Friability |
| --- | --- | --- | --- | --- | --- | --- | --- | --- | --- | --- |
|  | [%] |  | [%w/v] | [mPa·s] | [EBC] | [mg/L] | [mg/L] | [mg/L] | [%] | [%] |
| Barley | 39 | 6.1 ± 0 | 81.2 ± 0.6 | 0.981 ± 0.005 | 6.3 ± 0.1 | 265.1 ± 63.9 | 846.5 ± 14.3 | 160.2 ± 3.7 | 86.7 ± 0.4 | 66.2 ± 0.1 |
| Barley | 45 | 6.1 ± 0 | 81.4 ± 1.1 | 0.9 ± 0.005 | 6.7 ± 0.2 | 42.5 ± 1.7 | 894.2 ± 7.5 | 180.4 ± 2 | 89.7 ± 0.1 | 86.1 ± 1.2 |
| Wheat | 39 | 6.2 ± 0 | 84.8 ± 0.2 | 1.212 ± 0.004 | 9.6 ± 0.3 | 63 ± 8.7 | 602.2 ± 9.9 | 73.4 ± 0.9 | 80.1 ± 0.1 |  |
| Wheat | 45 | 6.1 ± 0 | 85.5 ± 0.4 | 1.18 ± 0.004 | 11.7 ± 0.4 | 36 ± 5.4 | 742.6 ± 3.5 | 94.4 ± 1.8 | 81.5 ± 0.4 |  |
| Oat | 39 | 6 ± 0 | 59.9 ± 1.8 | 0.938 ± 0.012 | 23.9 ± 2.1 | 368.5 ± 5.2 | 472.8 ± 9.9 | 79 ± 1.3 |  |  |
| Oat | 45 | 5.8 ± 0 | 60.5 ± 0.5 | 0.938 ± 0.008 | 20.3 ± 2.1 | 297 ± 9.6 | 517.2 ± 11.1 | 102 ± 1.9 |  |  |

Table 2. Chemical analysis of worts produced from different malt grain sources (iso 63 °C, n = 3)

| Sample | S.D. | pH | Extract | Viscosity | Viscosity Adj. to 8,6% | FDA | β-Glucan | Arabinoxylan | Soluble Nitrogen | FAN |
| --- | --- | --- | --- | --- | --- | --- | --- | --- | --- | --- |
|  | [%] |  | [%w/v] | [mPa·s] | [mPa·s] | [%] | [mg/L] | [mg/L] | [mg/L] | [mg/L] |
| Barley | 39 | 6.2 ± 0.01 | 9.2 ± 0.03 | 1.526 ± 0.005 | 1.481 ± 0.004 | 90 ± 0.4 | 154 ± 7.4 | 674.6 ± 124.8 | 862.4 ± 10.2 | 170.5 ± 1.1 |
| Barley | 45 | 6.1 ± 0.01 | 9.5 ± 0.04 | 1.477 ± 0.003 | 1.421 ± 0.001 | 93.7 ± 0.13 | 18.1 ± 1 | 752.5 ± 16 | 921.5 ± 13.6 | 197.4 ± 4.5 |
| Wheat | 39 | 6.4 ± 0.01 | 9.4 ± 0.02 | 1.981 ± 0.003 | 1.847 ± 0.003 | 69.1 ± 0.12 | 49.9 ± 0.7 | 721.4 ± 23.4 | 620.5 ± 9.8 | 79.5 ± 2.5 |
| Wheat | 45 | 6.2 ± 0.01 | 9.6 ± 0.03 | 1.975 ± 0.025 | 1.815 ± 0.021 | 70 ± 0.04 | 34.8 ± 2.1 | 883.3 ± 51.5 | 776.7 ± 9.1 | 106.4 ± 1.6 |
| Oat | 39 | 6.1 ± 0.02 | 6.7 ± 0.04 | 1.627 ± 0.011 | 1.812 ± 0.026 | 70.5 ± 0.26 | 476.1 ± 14.4 | 182.3 ± 87.3 | 589.8 ± 35 | 87.4 ± 1.5 |
| Oat | 45 | 5.9 ± 0.01 | 6.8 ± 0.07 | 1.61 ± 0.014 | 1.782 ± 0.025 | 70.3 ± 0.26 | 410.7 ± 18.7 | 170.9 ± 84.4 | 622.9 ± 8.1 | 82 ± 24.6 |

Figure S1. Conformation ratio and apparent density behavior vs molar mass of laboratory produced worts from different grain sources (n = 3).

Figure S2. Principal component analysis loadings of molar mass and conformation parameters (AF4-MALS-DRI) positively correlated to FC.
